# The second highest chromosome count among vertebrates is observed in cultured sturgeon and is associated with genome plasticity

**DOI:** 10.1186/s12711-016-0194-0

**Published:** 2016-02-11

**Authors:** Miloš Havelka, Dmytro Bytyutskyy, Radka Symonová, Petr Ráb, Martin Flajšhans

**Affiliations:** Faculty of Fisheries and Protection of Waters, South Bohemian Research Center of Aquaculture and Biodiversity of Hydrocenoses, Research Institute of Fish Culture and Hydrobiology, University of South Bohemia in Ceske Budejovice, Zátiší 728/II, 389 25 Vodňany, Czech Republic; Faculty of Fisheries Sciences, Hokkaido University, 3-1-1 Minato, Hakodate, Hokkaido 041-8611 Japan; Research Institute for Limnology, University of Innsbruck, Mondseestraße 9, 5310 Mondsee, Austria; Laboratory of Fish Genetics, Institute of Animal Physiology and Genetics, Czech Academy of Sciences, 277 21 Liběchov, Czech Republic

## Abstract

**Background:**

One of the five basal actinopterygian lineages, the Chondrostei, including sturgeon, shovelnose, and paddlefish (Order Acipenseriformes) show extraordinary ploidy diversity associated with three rounds of lineage-specific whole-genome duplication, resulting in three levels of ploidy in sturgeon. Recently, incidence of spontaneous polyploidization has been reported among cultured sturgeon and it could have serious negative implications for the economics of sturgeon farming. We report the occurrence of seven spontaneous heptaploid (7n) Siberian sturgeon *Acipenser baerii*, which is a functional tetraploid species (4n) with ~245 chromosomes. Our aims were to assess ploidy level and chromosome number of the analysed specimens and to identify the possible mechanism that underlies the occurrence of spontaneous additional chromosome sets in their genome.

**Results:**

Among 150 specimens resulting from the mating of a tetraploid (4n) *A. baerii* (~245 chromosomes) dam with a hexaploid (6n) *A. baerii* (~368 chromosomes) sire, 143 displayed a relative DNA content that corresponds to pentaploidy (5n) with an absolute DNA content of 8.98 ± 0.03 pg DNA per nucleus and nuclear area of 35.3 ± 4.3 μm^2^ and seven specimens exhibited a relative DNA content that corresponds to heptaploidy (7n), with an absolute DNA content of 15.02 ± 0.04 pg DNA per nucleus and nuclear area of 48.4 ± 5.1 μm^2^. Chromosome analyses confirmed a modal number of ~437 chromosomes in these heptaploid (7n) individuals. DNA genotyping of eight microsatellite loci followed by parental assignment confirmed spontaneous duplication of the maternal chromosome sets via retention of the second polar body in meiosis II as the mechanism for the formation of this unusual chromosome number and ploidy level in a functional tetraploid *A. baerii*.

**Conclusions:**

We report the second highest chromosome count among vertebrates in cultured sturgeon (~437) after the schizothoracine cyprinid *Ptychobarbus dipogon* with ~446 chromosomes. The finding also represents the highest documented chromosome count in Acipenseriformes, and the first report of a functional heptaploid (7n) genome composition in sturgeon. To our knowledge, this study provides the first clear evidence of a maternal origin for spontaneous polyploidization in cultured *A. baerii*. To date, all available data indicate that spontaneous polyploidization occurs frequently among cultured sturgeons.

**Electronic supplementary material:**

The online version of this article (doi:10.1186/s12711-016-0194-0) contains supplementary material, which is available to authorized users.

## Background

It is generally considered that the ancestral vertebrate genome underwent two rounds (1R and 2R) of whole-genome duplication (WGD) events [1–3] and that teleost fishes underwent an additional teleost-specific round of WGD (3R or TSGD) [4, 5]. Moreover, additional WGD events occurred independently in several teleostean lineages, e.g. 4R or SaGD in salmonids [6]. For most of the other vertebrates, there was no additional WGD since the 1R and 2R events [7, 8].

As a result of multiple rounds of lineage-specific WGD [9], the widest range of chromosome numbers among vertebrates is found in extant Acipenseriformes, which constitute an ancestral lineage of non-teleost ray-finned fishes [10]. Currently, three groups of acipenseriform species can be identified based on chromosome number, DNA content, and nucleus/cell size. The chromosome number reaches ~120, ~240, or ~360 [10, 11], and the genome size ranges from 2.44 in *Huso huso* [12] to 13.78 pg DNA per nucleus in *Acipenser brevirostrum* [13]. An increase in chromosome number is inherently associated with an increase in DNA content in the cell nucleus [14, 15], but an increase in nucleus/cell volume seems to correlate more with an increase in chromosome number than in DNA content [7, 16, 17].

Recent investigations were based on two scales of ploidy level in Acipenseriformes: (1) an evolutionary scale, which assumes tetraploid (4n)—octaploid (8n)—dodecaploid—(12n) relationships [18] and refers to ancient ploidy levels; and (2) a functional scale, which assumes diploid (2n)—tetraploid (4n)—hexaploid (6n) relationships [19] that originate from significant functional genome re-diploidization during the evolution of sturgeon [20, 21]. For clarity, in this study we relate all ploidy levels to the functional scale.

Genome plasticity in sturgeons, which display different ploidy levels and various chromosome numbers, combined with the ease with which different sturgeon species that differ in chromosome number can hybridize, result in hybrid individuals that have intermediate karyotypes compared to that of the parental species [22]. Moreover, hybridization can occur again between these hybrids and pure species [23].

Sturgeons are propagated in aquaculture, mainly for the production of black caviar and boneless meat. The high commercial value of sturgeon and the status of the wild sturgeon populations classified as critically endangered are conflicting issues. Overexploitation of wild populations for over 40 years has led to the listing of all sturgeon species in the Appendices to CITES (Convention on International Trade in Endangered Species of Wild Fauna and Flora) and also to the development of sturgeon aquaculture, originally for reintroduction, but more recently for caviar production [24]. Today, sturgeon farming is a rapidly growing branch of aquaculture, with China recognized as the leader in meat and caviar production, followed by Italy, France, Russia, and the USA [25]. To meet market demand for sturgeon products, aquaculture techniques make continuous progress, and commercial farms are increasingly using cultured broodstock.

The occurrence of sturgeon individuals with spontaneous modifications of ploidy levels, and hence DNA content, nucleus/cell size, and atypical chromosome numbers, has been reported in cultured sturgeon [14, 26–32]. Clearly, Acipenseriformes have a high tolerance for hybridization as well as for spontaneous doubling of chromosome sets (autopolyploidization). While the role of hybridization has been thoroughly explored in breeding of farm animals [33–35], including sturgeon [24, 35, 36], little research has been conducted on the influence of spontaneous polyploidy in cultured sturgeon.

In this study, we report the occurrence of seven Siberian sturgeon (*Acipenser baerii*) individuals with spontaneous heptaploidy (7n), *A. baerii* being a functional tetraploid species (4n) with ~245 chromosomes. These individuals originated from artificial crossbreeding between a hexaploid (6n ~368 chromosomes) *A. baerii* sire that was confirmed to be of spontaneous polyploid origin [31] and tetraploid (4n) *A. baerii* dam. The genetic predisposition of sturgeons for spontaneous polyploidization must be considered in aquaculture since it may represent a much more serious problem for sturgeon farming than currently believed. Since *A. baerii* is the most commonly cultured sturgeon species [24], our investigation is relevant to sturgeon aquaculture worldwide. The primary aims of our study were to assess ploidy level and chromosome number of assumed heptaploid (7n) *A. baerii* individuals and to identify the possible mechanisms that are responsible for the occurrence of spontaneous additional chromosome sets in their genome.

## Methods

### Sampling

This study was carried out in accordance with the Czech Law 246/1992 on animal welfare, for which the authors possess a certificate according to §17 of the law. Protocols underwent ethical review by the University of South Bohemia animal care committee (PP3/FROV/2012) and were approved by the University of South Bohemia animal care committee. Prior to handling, fish were anesthetized with 0.6 mL/L 2-phenoxyethanol (Merck Co., Darmstadt, Germany).

One-hundred-fifty specimens (with a mean body weight of 546 ± 78 g) that originated from artificial crossbreeding between a tetraploid (4n ~245 chromosomes) *A. baerii* dam with a hexaploid (6n ~368 chromosomes) *A. baerii* sire were examined in 2012. Originally, crossbreeding was conducted to confirm fertility/sterility of the hexaploid (6n) *A. baerii* sire. Details on the parental fish and crossbreeding experiments are in Havelka et al. [31]. Peripheral blood was collected from the caudal vessel into a heparinized syringe [37]. Fin clips were taken from parental fish as well as from the 150 progeny and stored in 96 % molecular grade ethanol.

### Flow cytometry

After sampling, 20 µL of blood was added to 1 mL of physiological solution and kept at 4 °C. Fifteen µL of this mixture were added to a 1 mL kit containing 4′,6-diamidino-2-phenylindole (DAPI; Partec GmbH, Görlitz, Germany). Blood from the diploid (2n) *A. ruthenus* was included as a standard in the same proportions for each sample. Samples were filtered through 30 μM nylon filters, maintained at ambient temperature in the dark for 15 min, and run on a CyFlow Cube 8 flow cytometer (Partec GmbH, Görlitz, Germany) using UV–LED light. Results were visualized by a single-parameter histogram that shows the relative fluorescence of the unknown sample and the standard. Ploidy level of each specimen was verified with respect to relative DNA content in erythrocyte nuclei. Results were presented as the mean of at least 3000 nuclei.

### Feulgen image analysis

Feulgen image analysis densitometry was used for the quantification of absolute DNA. Blood smear slides were prepared using the flame tip method [38]. Diploid (2n) and induced triploid (3n) *Tinca tinca* (DNA content 2.02 and 3.10 pg DNA per nucleus, respectively) were used as internal standards [39]. Samples were stained using a DNA staining kit following Feulgen (Merck Co., Darmstadt, Germany). Feulgen image analysis densitometry as described in Hardie et al. [38] was conducted using a 3CCD Sony DXC-9100P camera coupled to an Olympus BX50 microscope (objective 100×) with OLYMPUS MICROIMAGE v. 4.0 image analysis software package (Olympus Corp., Tokyo, Japan) to measure integrated optical density (IOD) and area of erythrocyte nuclei. The IOD of 10 fish per ploidy level and a minimum of 100 nuclei per specimen were measured and compared to the IOD of two standards in order to calculate genome size. The erythrocyte nuclear area was also assessed in 10 fish per ploidy level and minimum of 100 nuclei per specimen.

### Karyotyping

To verify the results on genome size, two individuals that were identified as heptaploid (7n) by cytometric examination were karyotyped. Metaphase chromosomes were prepared from peripheral blood leucocytes according to the protocol of Fujiwara et al. [40] and as described by Havelka et al. [31]. Representative Giemsa-stained metaphase chromosome plates were examined using an Olympus AX 70 microscope and recorded with an Olympus DP30VW digital camera. Well-spread metaphase chromosomes were arranged in karyotypes using Ikaros MetaSystems (Metasystems, Germany) software for chromosome quantification.

### Microsatellite DNA genotyping

Microsatellite genotyping was performed to determine the origin of the additional sets of chromosomes that were observed in heptaploid (7n) individuals. Genomic DNA was extracted from fin clips of parental fish, seven progeny that were presumed to be heptaploid (7n), and seven randomly chosen specimens indicated as pentaploid (5n) by cytometric examination. After testing 14 microsatellite markers, the following eight markers, AciG 35 [41], Afu 68 [42], AfuG 54, AfuG 135 [43], Aox 45 [44], Spl 101, Spl 163, and Spl 173 [45] were selected based on the level of polymorphism between parents. Amplification was carried out according to the protocol described by Havelka et al. [21]. Fragment analysis for these microsatellites was performed on a 3500 ABI Genetic Analyzer (Applied Biosystems, TM) using a GeneScan LIZ 600 fluorescent size standard (Applied Biosystems, TM), and genotypes were scored with the GeneMapper v.5.0 software (Applied Biosystems, TM).

### Origin of the spontaneous polyploidy

The origin of the spontaneous increase in ploidy level and chromosome number that was observed in the heptaploid (7n) individuals was investigated according to a slightly modified protocol reported by Gille et al. [32]. We determined the ratio of private dam and/or sire microsatellite alleles between the heptaploid (7n) individuals and their pentaploid (5n) full siblings. If an allele was unique to the sire or the dam, it was coded 1. Alleles present in both parental genotypes were not taken into account, since they were not informative for the analysis. All genotypes of the analysed progeny were assessed by this approach, and the number of dam and sire private alleles was determined for each microsatellite locus and each individual under study. The basic dataset included the number of private sire and dam microsatellite alleles at each locus in the seven heptaploid (7n) individuals and their pentaploid (5n) full siblings (see Additional file 1: Table S1). Subsequently, the total number of private sire and dam microsatellite alleles at all loci was calculated for each ploidy group and the following hypotheses were tested: (1) there is an increase in number of private dam alleles in the heptaploid (7n) individuals compared to their pentaploid (5n) full siblings, which means that the part of the genome that was duplicated originated from the dam; (2) there is an increase in number of private sire alleles in the heptaploid (7n) individuals compared to their pentaploid (5n) full siblings, which means that the part of the genome that was duplicated originated from the sire; and (3) there is an increase in number of private sire and dam alleles in the heptaploid (7n) individuals compared to their pentaploid (5n) full siblings, which means that the part of the genome that was duplicated originated from both parental individuals. The significance of the increase in number of dam and/or sire private microsatellite alleles between the heptaploid (7n) individuals and their pentaploid (5n) full siblings was tested by paired Student’s *t* test using the software Statistica v. 12 [46]. Prior to conducting the analysis, the assumption of normally distributed paired differences was examined by Shapiro–Wilk’s test, which showed that the data were normally distributed. The paired Student’s t test was performed separately for dam and sire private alleles. The number of private alleles was set as a measurement variable, and locus and ploidy were set as nominal variables. Each locus had one pair of observations for the measurement variable, one for number of private alleles in the heptaploid (7n) individuals and one for number of private alleles in their pentaploid (5n) full siblings. The level of significance was set at 0.05.

## Results

We used a variety of methods for ploidy determination and analyzed absolute DNA content and erythrocyte nuclear area for 150 fish. While 143 specimens displayed relative DNA contents that corresponded to pentaploidy (5n) with an absolute DNA content of 8.98 ± 0.03 pg DNA per nucleus and nuclear area of 35.3 ± 4.3 μm^2^, seven specimens exhibited relative DNA contents that corresponded to heptaploidy (7n), with an absolute DNA content of 15.02 ± 0.04 pg DNA per nucleus and nuclear area of 48.4 ± 5.1 μm^2^.

**Fig. 1:**
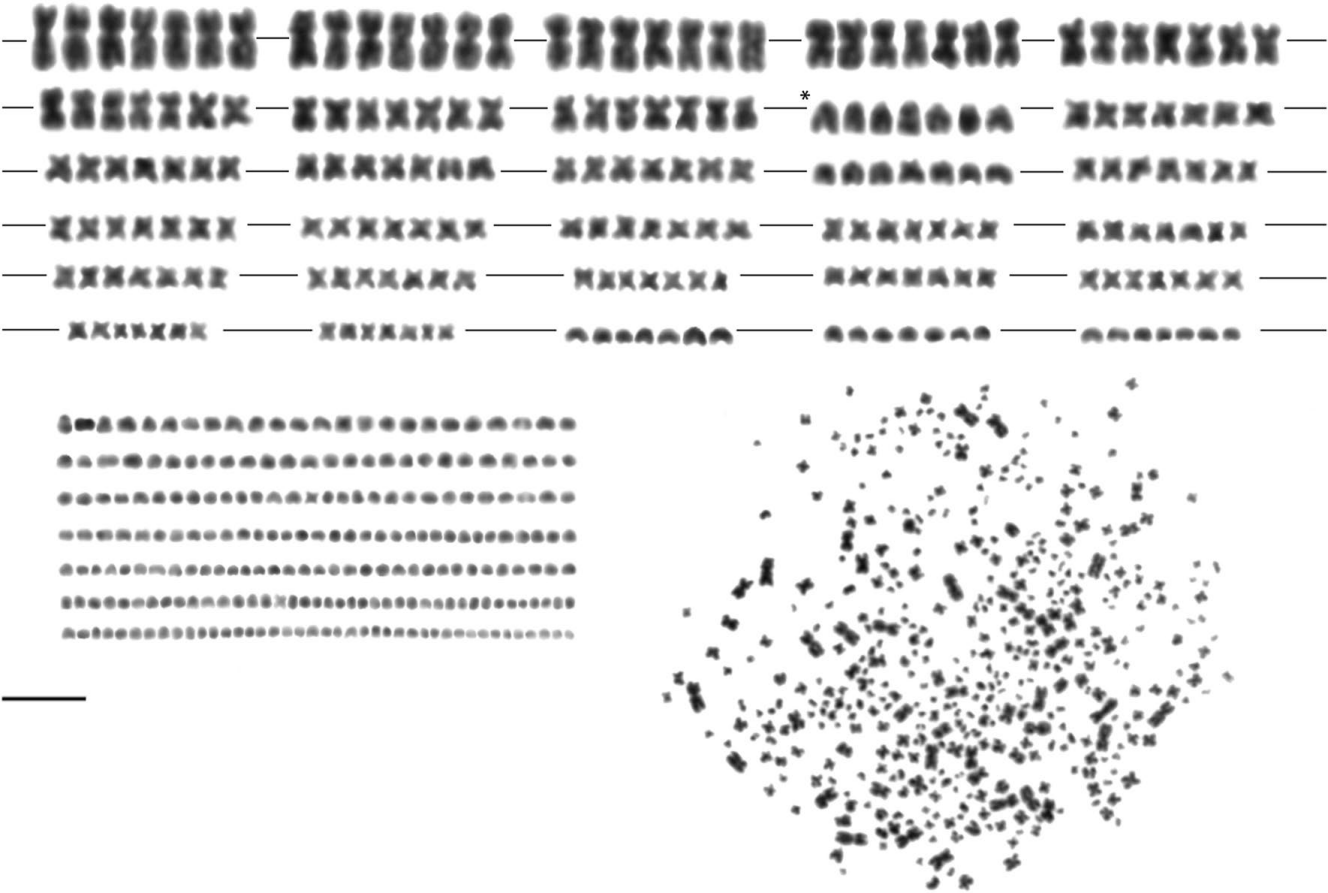
Metaphase chromosome spread and corresponding karyotype derived from Giemsa-stained chromosomes of heptaploid Siberian sturgeon *Acipenser baerii.* This specimen has 437 chromosomes; asterisks denote acipenserine cytotaxonomic markers, the group with the largest acrocentric chromosomes; *bar* is equivalent to 10 µm

Chromosome analyses confirmed that the number of chromosomes in the heptaploid (7n) individuals agrees with the level of ploidy. Nine countable metaphase chromosome spreads were analysed, with chromosome numbers ranging from 413 to 454 with a mean of 434. If the upper and lower extremes of the range of chromosome counts were omitted, a modal chromosome number of 430 ± 10 was found. This was demonstrated by analyzing a representative karyotype with 437 chromosomes from a heptaploid (7n) individual (Fig. 1). With the exception of the microchromosomes, all chromosomes could be grouped into heptaplets (Fig. 1). The recorded variation in total chromosome number was mainly due to variation in the number of small microchromosomes counted and to artefacts in the preparation (Fig. 1).

**Fig. 2:**
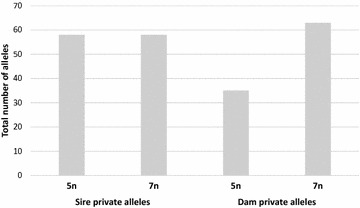
Comparison of total number of private dam and sire microsatellite alleles in the pentaploid (5n) and heptaploid (7n) analysed individuals. See also Additional file 1: Table S1

We found an unexpected increase in ploidy level, and hence genome size and chromosome number, in the genome of the heptaploid (7n) individuals. Microsatellite genotyping and subsequent parental assignment showed a significant increase in number of private dam alleles in the heptaploid (7n) individuals compared to their pentaploid (5n) full siblings (Paired Student t test, t = −11.2, 7 degrees of freedom, P = 0.00001). Conversely, there was no significant increase in number of private sire alleles between pentaploid (5n) and heptaploid (7n) full siblings (Paired Student t test, t = 0, 7 degrees of freedom, P = 1, see Fig. 2) The total number of private dam alleles was equal to 35 in the pentaploid (5n) group and 63 in the heptaploid (7n) group, which represents a 1.8-fold spontaneous increase (see Additional file 1: Table S1).

## Discussion

Due to the uniqueness and rarity of the heptaploid (7n) individuals and to prevent loss of live individuals, only two were used for karyotyping. Metaphase chromosome spreads were prepared from cultured leukocytes that were obtained from blood samples. In accordance with Hardie and Hebert [13], since DNA contents were similar for all seven heptaploid (7n) individuals, we assumed that they had similar chromosome numbers.

### Origin of known spontaneous polyploids

**Fig. 3:**
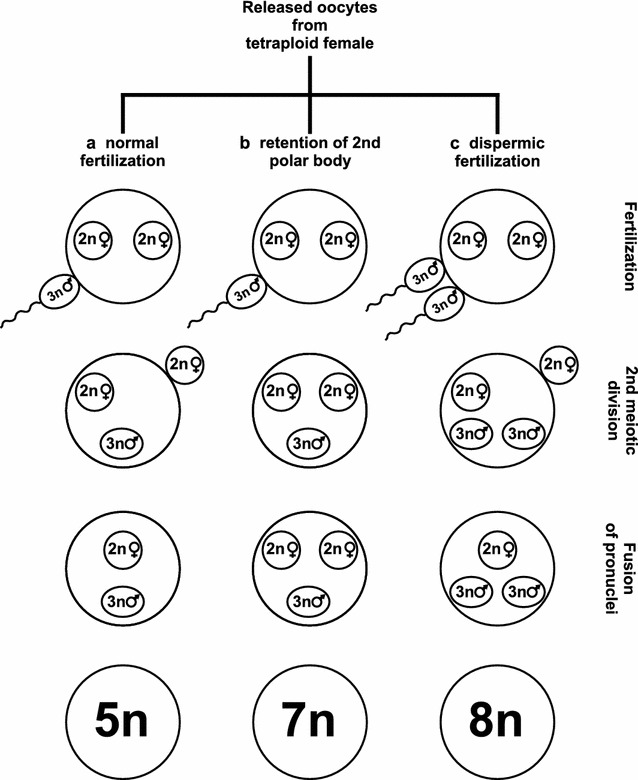
Comparison of normal fertilization, retention of the second polar body, and dispermic fertilization of a tetraploid female (4n) by spermatozoa of hexaploid male (6n). **a** Normal fertilization of diploid (2n) oocyte by triploid (3n) spermatozoon. **b** Retention of the second polar body in meiosis II results in tetraploid (4n) unreduced oocytes. If such an oocyte is fertilized by a triploid (3n) spermatozoon, it results in heptaploidy (4n^♀^ + 3n^♂^ = 7n), which is the ploidy level observed in the seven individuals under study. **c** Dispermic fertilization results in an octaploid (8n) zygote. If a diploid (2n) oocyte is fertilized by two triploid (3n) spermatozoa from a hexaploid male (6n), it will lead to octaploidy (2n^♀^ + 3n^♂^ + 3n^♂^ = 8n)

In this study, we report the second highest chromosome count among vertebrates after the schizothoracine cyprinid *Ptychobarbus dipogon* that has ~446 chromosomes [47] (see Additional file 2: Table S2). We showed that this very large number of chromosomes originates from spontaneous polyploidization. Generally, spontaneous polyploidization occurs via chromosome doubling, or production of unreduced gametes, or polyspermy. In animals, it is generally assumed to result from unreduced gamete formation [48, 49]. Among fish, production of unreduced oocytes via spontaneous duplication of the maternal chromosome set (SDM) is not rare [6]. Another mechanism of spontaneous polyploidy in fishes is the occurrence of polyspermic fertilization, which due to the presence of multiple micropyles in the oocytes of sturgeon, is theoretically more likely in this species compared to other fish taxa [50]. Our results confirmed that the observed increase in ploidy level, genome size, and chromosome number originated from the maternal genome by SDM. Because the *A. baerii* sire used in the experimental crossbreeding was hexaploid (6n) and thus, had triploid (3n) spermatozoa [31], dispermic or polyspermic fertilization can be excluded as the mechanism responsible for the observed heptaploidy in the 7n individuals (Fig. 3). This, together with the significant increase in private dam alleles that was found for the heptaploid (7n) specimens, provides conclusive evidence that the heptaploid (7n) fish resulted from the fertilization of unreduced oocytes with SDM from a tetraploid (4n) female by triploid (3n) spermatozoa from the hexaploid (6n) male.

Several mechanisms can explain SDM including apomixis, premeiotic endomitosis, and retention of the second polar body in meiosis II. Apomixis and premeiotic endomitosis have been shown to provide unreduced clonal oocytes that are genetically identical to the dams [51–54]. Because the microsatellite genotypes of the spontaneous heptaploid (7n) individuals that were analyzed in this study were not identical to those of the dam (see Additional file 1: Table S1), both apomixis and premeiotic endomitosis can be excluded as the mechanisms of SDM during oocyte formation in this case. Because of the 1.8-fold spontaneous increase in private dam alleles and because genotypes of the heptaploid (7n) individuals were not fully identical to those of the dam (i.e. recombination occurred), retention of the second polar body is the most plausible mechanism to explain SDM in the oocytes and the resulting spontaneous heptaploid (7n) individuals. This agrees with the findings of Gille et al. [32] who identified spontaneous polyploid individuals in cultured *A. transmontanus* and suggested that this spontaneous autopolyploidy was most probably caused by failure of the segregation of the second polar body during meiosis II.

### Spontaneous polyploidy and its influence on sturgeon populations

Spontaneous polyploidy is a phenomenon that has been observed in a number of cultured fish species [55] including *Oncorhynchus mykiss* [56], *Tinca tinca* [57, 58], *Anguilla japonica* [59], *Oncorhynchus kisutch* [60], *Silurus glanis* [61], and *Salmo salar* [62], as well as in cultured sturgeon, e.g. hybrid (bester) sturgeon (*H. huso* × *A. ruthenus*) [26], *A. ruthenus* [30], *A. baerii* [31], *A. transmontanus* [27, 29, 32], *A. gueldenstaedtii* [15], *H. dauricus* and *A. mikadoi* [14]. In diploid (2n) species, the presence of an additional set of chromosomes results in triploid (3n) individuals that are infertile or sub-sterile. In contrast, spontaneous polyploidization in tetraploid (4n) sturgeon species results in fertile hexaploid (6n) individuals, as reported for *A. baerii* [31] and *A. transmontanus* [29, 32]. Backcrossing of these spontaneous hexaploids (6n) to tetraploid (4n) individuals produces fully viable pentaploid (5n) progeny [29, 31, 32]. Although the reproductive potential of such pentaploid (5n) individuals is not confirmed, they are likely to present a significantly reduced fertility, since their chromosomes cannot pair during the zygotene stage of meiosis prophase I, due to the odd number of chromosome sets. Such impairment interferes with gonad development and gametogenesis, which is similar to what is observed for triploid (3n) individuals [63]. Currently, most cultured sturgeons originate from tetraploid (4n) species. The occurrence of fertile spontaneous polyploidy individuals among a tetraploid (4n) broodstock can negatively affect its reproductive capacity, and thus caviar production and the overall efficiency of sturgeon farms.

Prolongation of the period between ovulation, stripping, and fertilization may increase the incidence of retention of the second polar body in sturgeon artificial reproduction conditions [26, 32], as was observed for *O. mykiss* [56], *T. tinca* [58], and *A. japonica* [59]. To reduce the incidence of spontaneous polyploidy in cultured sturgeon and also in order to eliminate restocking of spontaneous polyploidy individuals into the wild populations, eggs should be stripped and fertilized immediately after ovulation, and the ploidy level of all fish should be determined before their inclusion in reproduction or reintroduction programs.

A maternal genetic predisposition for producing unreduced oocytes was suggested in *O. mykiss* [64, 65], *C. carpio* [66, 67], *T. tinca* [57], and *Misgurnusanguillicaudatus* [68], and was also discussed by Gille et al. [32] for *A. transmontanus.* Because polyploidization provides some genetic advantages [69], such genetic predisposition for the formation of unreduced gametes may be conserved in the genome of spontaneous polyploidy individuals and transmitted to the progeny, as was recently hypothesized by Mason and Pires [49]. This suggests that a higher incidence of spontaneous polyploidy may occur in cultured sturgeon than previously supposed, and hence could represent a greater issue for aquaculture.

The impact of spontaneous polyploidy on wild sturgeon populations has not been investigated. In plants, spontaneous polyploidization or production of unreduced gametes is known to occur in wild populations and the resulting individuals are able to survive and compete successfully in or near sites occupied by diploid populations [70]. Spontaneous polyploid individuals have also been observed in wild populations of amphibians, but at a very low frequency and the significance of this phenomenon for wild populations remains unclear [71]. In fish, spontaneous polyploidization has been reported in wild populations of several species e.g. [51, 52, 54], but the phenomenon appears to be a natural characteristic of a given species/population and its reproductive biology, rather than an unusual event [55]. To our knowledge, there is no evidence in the literature on the incidence of spontaneous polyploidization in wild sturgeon populations. However, sturgeon gametes can be exposed to stresses from unstable and rapidly fluctuating environmental conditions in natural spawning habitats, resulting in chromosome doubling, as suggested for fish in general [7]. In addition, since spontaneous polyploidy individuals have been reported in farmed populations and since reintroduction programs generate considerable interest, the presence of such spontaneous polyploid individuals that originate from release programs, is likely in wild sturgeon populations. Thus, ecological interactions may occur between spontaneous polyploids and wild individuals. As in captive sturgeon populations, fertile spontaneous polyploid individuals can spawn in the nature and result in progeny with reduced fertility. It may lead to a significant decrease in fitness of wild populations and thus have a negative impact on this critically endangered sturgeon species that has a very low abundance of natural spawners. Therefore, this is an important issue for reintroduction programs of this endangered sturgeon species that should be addressed in future studies.

Finally, formation of unreduced gametes has recently been suggested as a mechanism of speciation, contrary to the general interpretation that it is an evolutionary mishap [49]. It is interesting to note that most polyploid fish belong to the lower ray-finned fish and display a high incidence of hybridization [55]. Therefore, we hypothesize that the capacity of the sturgeon species for spontaneous polyploidization may contribute to explain their “living fossil” status, slow rate of genome evolution [72] and tolerance for hybridization.

## Conclusions

This is the first study that describes a heptaploid (7n) genome composition in sturgeon based on seven individuals of *A. baerii*. It represents the highest documented chromosome count in Acipenseriformes and the second highest among all vertebrates. Spontaneous duplication of the maternal chromosome sets via retention of the second polar body in meiosis II was confirmed as the mechanism that underlies the formation of this high ploidy level and chromosome count. To the best of our knowledge, this represents the first evidence for a maternal origin of spontaneous polyploidization in *A. baerii*.

## Additional files

10.1186/s12711-016-0194-0 Genotyping results with highlighted private dam and sire microsatellite alleles observed at all analysed loci for parental individuals, and for pentaploid (5n) and heptaploid (7n) full siblings.
10.1186/s12711-016-0194-0 Species with the highest chromosome count in vertebrate families and their nuclear DNA content. Asterisks denote references to DNA content.
