# Supplementary material for: The second highest chromosome count among vertebrates is observed in cultured sturgeon and is associated with genome plasticity
Source: Genet Sel Evol. 2016 Feb 11;48:12. doi: 10.1186/s12711-016-0194-0 (PMC4751722; doi:10.1186/s12711-016-0194-0)
Supplement: Supplementary file 2 — 10.1186/s12711-016-0194-0 Species with the highest chromosome count in vertebrate families and their nuclear DNA content. Asterisks denote references to DNA content. [file 12711_2016_194_MOESM2_ESM.pdf]

Species	Family	DNA content (pg/nuclei)	Chromosome number	References
**Actinopterygii**				
Ptychobarbus dipogon	Cyprinidae	-	446	[1]
Acipenser baerii	Acipenseridae	15.02	437	present study
Acipenser mikadoi	Acipenseridae	12.9	402	[2*, 3]
Acipenser brevirostrum	Acipenseridae	13.07	372	[4, 5*]
Acipenser baerii	Acipenseridae	12.7	368	[6*, 7]
Acipenser transmontanus	Acipenseridae	9.46	271	[5*, 8]
Huso dauricus	Acipenseridae	8.3	268	[9*, 10]
Acipenser schrenckii	Acipenseridae	8.2	266	[9*, 10]
Carassius auratus langsdorfii	Cyprinidae	-	204	[11]
Carassius auratus auratus	Cyprinidae	5.12	150	[12]
Corydoras aeneus	Callichthyidae	6.6	134	[13]
**Myxini**				
Eptatretus burgeri	Myxinidae	6.0	36	[14*, 15]
Paramyxine atami	Myxinidae	-	34	[14]
**Petromyzontida**				
Geotria australis	Geotriidae	3.08	180	[16*, 17]
Lethenteron reissneri	Petromyzontidae	-	174	[18]
**Chondrichthyes**				
Notorynchus cepedianus	Notorynchidae	8.8	104	[19]
Heterodontus francisci	Heterodontidae	17.5	102	[19]
**Sarcopterygii**				
Protopterus dolloi	Protopteridae	163.2	68	[20]
Latimeria chalumnae	Latimeriidae	7.2	48	[21*, 22]
**Amphibia**				
Xenopus longipes	Pipidae	-	108	[23]
Xenopus ruwenzoriensis	Pipidae	7.95	108	[24*, 25]
**Reptilia**				
Platemys platycephala	Chelidae	-	96	[26]
Carettochelys insculpta	Carettochelyidae	-	68	[27]
**Aves**				
Alcedo atthis	Alcedinidae	2.8	138	[28, 29*]
Upupa epops	Upupidae	2.56	126	[28, 30*]
**Mammalia**				
Tympanoctomys barrerae	Octodontidae	16.8	102	[31, 32*]
Ichthyomys pittieri	Cricetidae	-	92	[33]

References

1. Yu XY, Yu XJ. A schizothoracin fish species, Diptychus dipogon, with very high number of chromosomes Chrom Inform Serv. 1990;48:17-8.
2. Zhou H, Fujimoto T, Adachi S, Yamaha E, Arai K. Genome size variation estimated by flow cytometry in Acipenser mikadoi, Huso dauricus in relation to other species of Acipenseriformes. J Appl Ichthyol. 2011;27:484-91.
3. Zhou H, Fujimoto T, Adachi S, Abe S, Yamaha E, Arai K. Molecular cytogenetic study on the ploidy status in *Acipenser mikadoi*. J Appl Ichtyol. 2013;29:51-5.
4. Kim DS, Nam YK, Noh JK, Park CH, Chapman FA. Karyotype of North American shortnose sturgeon Acipenser brevirostrum with the highest chromosome number in Acipenseriformes. Ichthyol Res. 2005;52:94-7.
5. Blacklidge KH, Bidwell CA. Three ploidy levels indicated by genome quantification in Acipenseriformes of North America J Hered. 1993;84:427-30.
6. Bytyutskyy D, Srp J, Flajšhans M. Use of Feulgen image analysis densitometry to study the effect of genome size on nuclear size in polyploid sturgeons. J Appl Ichthyol. 2012;28:704-8.
7. Havelka M, Hulák M, Ráb P, Rábová M, Lieckfeldt D, Ludwig A, Rodina M, Gela D, Pšenička M, Bytyutskyy D, Flajšhans M. Fertility of a spontaneous triploid Siberian sturgeon, *Acipenser baerii.* BMC Genetics. 2014;15:5.
8. Van Eenennaam AL, Murray JD, Medrano JF. Mitotic analysis of the North American white sturgeon, Acipenser transmontanus Richardson (Pisces, Acipenseridae), a fish with a very high chromosome number. Genome. 1998;41:266-71.
9. Zhou H, Fujimoto T, Adachi S, Yamaha E, Arai K. Genome size variation estimated by flow cytometry in Acipenser mikadoi, Huso dauricus in relation to other species of Acipenseriformes. J Appl Ichthyol. 2011;27:484-91.
10. Vasil'ev VP, Vasil'eva ED, Shedko SV, Novomodny GV. How many times has polyploidization occurred during acipenserid evolution? New data on the karyotypes of sturgeons (Acipenseridae, Actinopterygii) from the Russian Far East. J Ichthyol. 2010;50:950-9.

11. Kobayasi H, Kawashima Y, Takeuchi N. Comparative chromosome studies in the genus Carassius, especially with a finding of polyploidy in the ginbuna (C. auratus langsdorfii). Japan J Ichthyol. 1970;17:153-60. (in Japanese with English abstract).

12. Suzuki A, Taki Y, Mochizuki M, Hirata J. Chromosomal speciation in Eurasian and Japanese Cyprinidae (Pisces, Cypriniformes) Cytobios. 1995;83:171-86.

13. Turner BJ, Diffoot N, Rasch EM. The callichtyid catfish Corydoras aeneus is an unresolved diploid-tetraploid sibling species complex. Ichtyological Exploration of Freshwaters. 1992;3:17-23.

14. Nakai Y, Kubota S, Kohno S. Chromatin diminution and chromosome elimination in four Japanese hagfish species. Cytogenet Cell Genet. 1991;56(3-4):196-8.

15. Kohno S, Nakai Y, Satoh S, Yoshida M, Kobayashi H. Chromosome elimination in the Japanese hagfish, Eptatretus burgeri (Agnatha, Cyclostomata). Cytogenet Cell Genet. 1986;41:209-14.

16. Robinson ES, Potter IC, Atkin NB. The nuclear DNA content of lampreys. Experientia. 1975;31:912-3.

17. Robinson ES, Potter IC. The chromosomes of the Southern Hemispheric Lamprey, Geotria australis Gray. Experientia. 1981;37:239-240.

18. Sasaki M, Hitotsumachi S. Notes of the chromosomes of a fresh water lamprey Entosphenus ressnerii (Cyclostomata). Chrom Inform Serv. 1967;8:22-4.

19. Schwartz FJ, Maddock MB. Comparisons of karyotypes and cellular DNA contents within and between major lines of elasmobranch In: Uyeno T, Arai R, Tuniuchi T, Matsuura K, editors. Indo-Pacific Fish Biology. Tokyo, Japan: Ichthyological Society of Japan; 1986. p. 148-157.

20. Vervoort A. Tetraploidy in Protopterus (Dipnoi). Experientia. 1980;36:294-5.

21. Cimino MC, Bahr GF. The nuclear DNA content and chromatine ultrastructure of the coelacanth Latimeria chalumnae. Exp Cell Res. 1974;88:263-72.

22. Bogart JP, Balon EK, Bruton MN. The chromosomes of the living coelacanth and their remarkable similarity to those of one of the most ancient frogs. J Hered. 1994;85:322-5.

23. Loumont C, Kobel, HR. *Xenopus longipes* sp. nov., a new polyploid pipid from western Cameroon. Revue Suisse de Zoologie. 1991;98:731-8.

24. Thiébaud CH, Fischberg M. DNA content in the genus *Xenopus.* Chromosoma. 1977;59:253-7.

25. Tymowska J, Fischberg M. Chromosome complements of the genus *Xenopus*. Chromosoma. 1973;44:335-42.

26. Bull JJ, Legler JM. Karyotypes of side-necked turtles (Testudines, Pleurodira) Canadian J Zool. 1980;58:828-41.

27. Valenzuela N, Adams DC. Chromosome number and sex determination coevolve in turtles. Evolution. 2011;65-6:1808-13.

28. Bian X, Li Q. Studies on the Karyotypes of Birds. The 20 species of Climber birds (Aves). Zoological Research. 1989;10 (4): 309-17.

29. De Smet WHO, The nuclear Feulgen-DNA content of the vertebrates (especially reptiles), as measured by fluorescence cytophotometry, with notes on the cell and chromosome size. Acta Zoologica et Pathologica Antverpiensia. 1981;76:119-67.

30. Wright NA, Gregory TR, Witt CC. Metabolic 'engines' of flight drive genome size reduction in birds. Proc R Soc B. 2014;281:20132780.

31. Gallardo MH, González CA, Cebrián I. Molecular cytogenetics and allotetraploidy in the red vizcacha rat, *Tympanoctomys barrerae* (Rodentia, Octodontidae). Genomics. 2006;88 (2), 214-21.

32. Gallardo MH, Bickham JW, Honeycutt RL, Ojeda RA, Köhler N. Discovery of tetraploidy in a mammal. Nature. 1999;401:341.

33. Schmid M, Fernández-Badillo A, Feichtinger W, Steinlein C, Roman JI. On the highest chromosome number in mammals. Cytogenet Genome Res. 1988;49(4):305-8.
